# Urine-Based Approaches for Screening, Diagnosis, and Surveillance of Urothelial Carcinoma

**DOI:** 10.3390/jpm16030135

**Published:** 2026-02-28

**Authors:** Vladimir Bilim, Senji Hoshi

**Affiliations:** 1 Department of Urology, Kameda Daiichi Hospital, Niigata 950-0165, Japan; 2 Department of Urology, Division of Molecular Oncology, Graduate School of Medical and Dental Sciences, Niigata University, Niigata 951-8510, Japan; 3 Affiliated Long-Term Care Health Facility, Sendai South Hospital, Japan Community Healthcare Organization, Sendai 980-0022, Japan

**Keywords:** urothelial carcinoma, bladder cancer screening, molecular diagnostics, cancer surveillance, noninvasive diagnostics, urine cytology, urinary biomarkers, liquid biopsy, next-generation sequencing, cfDNA, exosomes, microRNA

## Abstract

**Background:** Urothelial carcinoma (UC) is characterized by high recurrence rates and the need for long-term surveillance. Cystoscopy remains the diagnostic gold standard but is invasive, costly, and burdensome for patients. Urine, as a tumor-proximal and non-invasive biospecimen, represents an attractive source for biomarkers enabling screening, diagnosis, risk stratification, and follow-up. **Objective:** This review summarizes current and emerging urine-based diagnostic approaches for UC, ranging from conventional cytology to advanced molecular technologies, and discusses their clinical utility, limitations, and future perspectives. **Methods:** A narrative review of the literature was conducted focusing on urine-based diagnostics for UC, including urinary cytology, FDA-approved and investigational protein and DNA/RNA biomarkers, next-generation sequencing (NGS), cell-free DNA (cfDNA), exosomes, and microRNAs. Evidence from clinical validation studies, meta-analyses, and translational research was evaluated. **Results:** Urinary cytology remains highly specific for high-grade disease but has limited sensitivity for low-grade tumors. Protein- and DNA-based biomarkers have improved sensitivity but often lack sufficient specificity for standalone use. Recent advances in NGS-based assays enable comprehensive detection of tumor-specific genomic alterations in urinary cfDNA, offering high sensitivity for both initial diagnosis and disease monitoring. Exosomes and microRNAs represent promising biomarkers reflecting tumor biology, though standardization and large-scale validation are ongoing challenges. Overall, multimodal approaches combining cytology with molecular assays appear most promising for clinical implementation. **Conclusions:** Urine-based diagnostics are rapidly evolving toward integrated liquid biopsy platforms capable of transforming UC management. While several assays show strong potential to reduce reliance on cystoscopy, robust prospective validation, cost-effectiveness analyses, and clinical integration strategies are required before widespread adoption.

## 1. Introduction

Urothelial carcinoma (UC), encompassing malignancies of the bladder and upper urinary tract, represents one of the most common cancers of the urinary system and poses a substantial clinical and economic burden worldwide. Bladder cancer (BC) alone ranks among the top ten most frequently diagnosed cancers globally, with particularly high incidence in industrialized countries [1]. A hallmark of UC is its biological heterogeneity and high recurrence rate, especially in non–muscle-invasive bladder cancer (NMIBC), necessitating lifelong surveillance in many patients [2,3]. As a consequence, UC is among the most costly malignancies to manage on a per-patient basis [4].

Cystoscopy remains the cornerstone of diagnosis and surveillance for UC. While highly informative, cystoscopy is invasive, uncomfortable, and resource-intensive, and its diagnostic accuracy may be limited for small papillary tumors or flat lesions such as carcinoma in situ (CIS) [2,4]. Imaging modalities, including computed tomography urography and magnetic resonance imaging, provide valuable staging information but lack sensitivity for early disease and are not suitable for frequent surveillance [5]. These limitations have driven sustained interest in non-invasive diagnostic alternatives.

Urine represents an ideal medium for UC diagnostics. As a tumor-proximal biofluid, urine contains exfoliated tumor cells, cell-free nucleic acids, proteins, metabolites, and extracellular vesicles shed directly from the urothelium [6]. Collection is simple, non-invasive, repeatable, and well accepted by patients. Historically, urine-based diagnostics relied primarily on cytological evaluation. However, rapid advances in molecular biology and genomic technologies have expanded the scope of urinary testing from morphological assessment to comprehensive molecular profiling [7].

NMIBC can be broadly stratified into low-grade papillary tumors with low progression risk which are frequently characterized by activating mutations in FGFR3, HRAS, PIK3CA, and high-grade tumors including CIS which carry a substantial risk of progression to muscle invasive bladder cancer (MIBC) and are commonly associated with alterations in TP53, RB1, ERCC2, and CDKN2A (Table 1). The most frequent genomic alterations in UC are summarized in Table 2.

The 2020 consensus molecular classification of MIBC further stratifies tumors into distinct molecular subtypes with characteristic genomic alterations, transcriptomic profiles, and clinical behaviors [8] (Table 3). Luminal Papillary (LumP) is characterized by FGFR3 mutations/fusions (FGFR3–TACC3), PIK3CA, KDM6A, and STAG2 mutations and has expression of KRT20, GATA3, UPK. Luminal Non-Specified (LumNS) is characterized by ELF3, PPARG mutations and ERBB2 (HER2) amplification. Luminal Unstable (LumU) is characterized by Genomic instability and High mutation burden. Key Alterations are in TP53, ERCC2, ERBB2, and DNA damage repair (DDR) genes. Luminal tumors have high expression of GATA3, CK20, and FOXA1. For Basal/Squamous (Ba/Sq) alterations in TP53, RB1, and NF1 genes are typical and STAT3 activation is frequently seen. High expression of KRT5, KRT14, and CD44 and low expression of GATA3 is usually seen. CK5/6+, CK14+, CD44+, p63+. Stroma-rich is characterized by TGF-β signaling and high expression of extracellular matrix genes. Neuroendocrine-like (NE-like) has alterations in TP53, RB1 (biallelic loss), MYCN, and AURKA genes. Expression of Synaptophysin, Chromogranin, and INSM1 is usually increased. These molecular distinctions provide important biological context for urine-based biomarker development and interpretation.

This review provides an in-depth and updated overview of urine-based diagnostic approaches in UC, spanning conventional urinary cytology, protein and DNA biomarkers, next-generation sequencing (NGS), urinary cell-free DNA (cfDNA), exosomes, microRNAs, and other non-coding RNAs. We discuss their biological basis, analytical performance, clinical applications, and limitations, with particular emphasis on their role in screening, diagnosis, risk stratification, and disease surveillance. Finally, we explore integrative strategies and future directions aimed at translating urine-based diagnostics into routine clinical practice.

## 2. Literature Search Strategy

This narrative review is based on a comprehensive review of the published literature using PubMed/MEDLINE, Embase, Scopus, Cochrane Library, and Web of Science. Searches were conducted for studies published between January 2000 and October 2025, with priority given to contemporary guidelines, systematic reviews, and clinically relevant original studies. Search terms included combinations of Medical Subject Headings (MeSH) and free-text terms such as “urothelial carcinoma,” “bladder cancer,” “upper tract urothelial carcinoma,” “urinary biomarkers,” “DNA methylation,” “circulating tumor DNA,” “NGS,” “exosomes,” and “microRNA.” Additional relevant studies were identified through manual review of reference lists.

## 3. Urinary Cytology

Urinary cytology is one of the oldest and most widely used non-invasive tests for UC. It involves microscopic examination of exfoliated urothelial cells obtained from voided urine, catheterized specimens, or bladder washings [9]. Cytology is particularly valued for its high specificity, often exceeding 90%, especially for high-grade UC including CIS [10]. Standard urinary cytology reports classify findings from Class I to V, with Classes IV and V considered positive for malignancy, while Class III is regarded as atypical/suspicious/equivocal and is frequently presented in the case of inflammation or urinary stone disease. The primary limitation of urinary cytology is its low sensitivity for low-grade tumors, which often exhibit minimal cytological atypia. Reported sensitivities for low-grade UC may be as low as 20–40%, whereas sensitivities for high-grade disease typically exceed 70% [10]. Diagnostic accuracy is influenced by sample quality, tumor shedding, and the experience of the cytopathologist, leading to interobserver variability. To address these challenges, the Paris System for Reporting Urinary Cytology (TPS) was introduced following international consensus, with the primary goal of improving standardization and focusing the diagnostic performance on the detection of high-grade urothelial carcinoma (HGUC) [11]. TPS defines seven diagnostic categories―Non-diagnostic/Inadequate, Negative for NHGUC, Atypical Urothelial Cells (AUC), Suspicious for HGUC (SHGUC), HGUC, Low-Grade Urothelial Neoplasia (LGUN), and Other―each associated with defined cytomorphological criteria and clinical implications [12]. By emphasizing nuclear atypia the system focuses on the detection of high-grade UC and discourages overinterpretation of atypical but benign findings. Adoption of the TPS has reduced the rate of equivocal diagnoses and improved reproducibility, though sensitivity limitations for low-grade disease persist. TPS is a standardized, evidence-based reporting framework developed to improve the consistency, reproducibility, and clinical usefulness of urinary cytology reports [13]. It was first introduced in 2016 following international consensus, with the primary goal of enhancing detection and classification of high-grade UC (HGUC) while reducing indeterminate interpretations [12].

TPS has since been widely adopted in clinical practices globally. It has an updated version (TPS 2.0) with refined criteria and additional guidance for ancillary testing [14]. TPS emphasizes urinary cytology’s strength in diagnosing high-grade lesions rather than low-grade disease because urine cytology has higher accuracy for high-grade cancers. Less emphasis is placed on reliably diagnosing low-grade UC. TPS was designed to reduce overdiagnosis, particularly of low-grade urothelial neoplasia, by shifting the focus of urinary cytology toward HGUC. It has been shown to reduce interobserver variability and improve reproducibility across institutions compared with pre-TPS reporting. While TPS improves specificity for high-grade urothelial carcinoma, it is associated with reduced sensitivity for low-grade tumors, resulting in a higher false-negative rate for LGUC. Reports on changes in false-positive rates have been variable across studies.

Despite its shortcomings, urinary cytology remains a key component of UC management. It is widely used in surveillance protocols, particularly for patients at high risk of progression. Rather than being replaced, cytology increasingly serves as a reference standard against which novel urinary biomarkers are compared and combined.

## 4. Urinary Biomarkers in Clinical Use

In an effort to overcome the sensitivity limitations of cytology, numerous urinary biomarkers have been developed and evaluated over the past several decades (Summarized in Table 4 and Table 5). Several dozens of urinary biomarkers have been reported for use in BC diagnosis, but only a few are approved by FDA and are commercially available [15]. These assays are broadly classified into protein-based and nucleic acids (DNA and RNA)-based tests and are primarily intended to detect tumor-associated molecules released into urine or in exfoliated tumor cells.

FDA-approved urinary biomarkers include nuclear matrix protein 22 (NMP22), bladder tumor antigen (BTA) assays, immunocytology (uCyt+), and fluorescence in situ hybridization (UroVysion FISH). More recently developed RNA-based assays such as Cxbladder and Xpert Bladder Cancer have gained clinical traction in selected settings, although regulatory status and guideline endorsement vary across regions [16].

Overall, these biomarkers demonstrate higher sensitivity than cytology, particularly for low-grade tumors, but often at the expense of reduced specificity. False-positive results are common in the presence of hematuria, inflammation, urinary tract infection, recent instrumentation, or intravesical therapy with BCG, limiting their widespread adoption as cystoscopy-sparing tools [16].

### 4.1. NMP22 Protein Assays

NMP22 was first described in 1974. NMP22 is a component of nuclear mitotic apparatus involved in DNA replication, transcription, and RNA processing. Although present in all nucleated cells, NMP22 is released in increased quantities into the urine from UC cells due to elevated cell turnover and apoptosis. Urinary NMP22 levels have been reported to be up to 20–25 times higher in patients with BC compared with individuals without malignancy [17]. Two commercial NMP22 assays are available: the qualitative point-of-care BladderCheck test and the quantitative laboratory-based NMP22 ELISA. BladderChek^®^ provides results within approximately 30 min and does not require intact cells, making it suitable for outpatient settings. NMP22 received FDA approval in 2000 as an adjunct to cystoscopy for BC detection.

Reported sensitivity of NMP22 ranges from 47% to 85%, generally exceeding that of cytology for low-grade tumors, while specificity ranges from 60% to 90%. Combination of NMP22 testing with cystoscopy has been shown to increase overall detection sensitivity compared with cystoscopy alone. However, false-positive results are frequently observed in benign conditions such as hematuria, urolithiasis, infection, and following intravesical therapy, limiting its specificity [18].

### 4.2. BTA Assays

Bladder tumor antigen assays detect complement factor H–related proteins produced by BC cells, which may protect tumor cells from complement-mediated lysis [19,20,21]. Two FDA-approved BTA tests are commercially available: BTA Stat, a qualitative point-of-care assay, and BTA TRAK, a quantitative laboratory-based ELISA [22].

Meta-analyses and systematic reviews report mean sensitivities of approximately 60–70% and specificities of 65–75% for both assays. While BTA assays demonstrate higher sensitivity than cytology, particularly for low-grade tumors, their specificity is substantially compromised by benign urological conditions, including hematuria, cystitis, urinary stones, and recent instrumentation. Consequently, BTA assays are not recommended as standalone diagnostic tools and are primarily used as adjunctive tests in selected clinical contexts.

### 4.3. uCyt+ (Formerly ImmunoCyt)

The uCyt+ assay (formerly ImmunoCyt) is a cell-based immunofluorescence test that uses monoclonal antibodies targeting tumor-associated antigens, including mucins (M344, LDQ10) and a high-molecular-weight glycosylated form of carcinoembryonic antigen (19A211), expressed on urothelial carcinoma cells [23]. The test is performed on exfoliated urothelial cells and is approved for use in BC surveillance. uCyt+ demonstrates higher sensitivity than cytology across tumor grades, including CIS, with reported sensitivities ranging from 70% to 85%. However, specificity is generally lower than cytology alone, and false-positive results may occur in the presence of inflammation or reactive atypia. As a result, uCyt+ is most commonly used in combination with cytology to improve overall diagnostic performance rather than as an independent test.

### 4.4. UroVysion

UroVysion FISH is a DNA-based, cell-based assay that detects chromosomal abnormalities commonly associated with UC, including aneuploidy of chromosomes 3, 7, and 17 and deletion of the 9p21 locus harboring CDKN2A and CDKN2B tumor suppressor genes [24]. The assay received FDA approval in 2005 and remains the most widely used molecular cytogenetic test for UC detection and surveillance.

Reported sensitivity of UroVysion ranges from 42% to 83% for non–muscle-invasive tumors and exceeds 90% for muscle-invasive disease, with particularly strong performance in high-grade tumors [25]. UroVysion has demonstrated utility in cases of equivocal or negative cytology and may predict recurrence earlier than cystoscopy in some patients. Limitations include higher cost, requirement for specialized laboratory infrastructure, and reduced specificity in inflammatory conditions.

### 4.5. RNA-Based Assays: Cxbladder and Xpert Bladder Cancer

Cxbladder is a quantitative RT-PCR–based urine assay measuring expression of five genes (MDK, HOXA13, CDC2, IGFBP5, and CXCR2) associated with BC [26]. The test is primarily used in patients presenting with hematuria and for surveillance of NMIBC. Reported sensitivity and specificity are approximately 80–85% and 80–90%, respectively, with particularly high specificity for high-grade tumors.

The Xpert^®^ Bladder Cancer assay measures urinary mRNA expression of a five-gene panel (ABL1, CRH, IGF2, UPK1B, and ANXA10) using automated RT-PCR technology. Sensitivity is reported at approximately 75–80% overall, with higher sensitivity for high-grade NMIBC compared with low-grade disease [27]. Both assays offer rapid turnaround times and standardized platforms but remain adjunctive tools pending broader guideline endorsement.

Collectively, FDA-approved urinary biomarkers improve sensitivity compared with cytology, particularly for low-grade tumors, but suffer from limited specificity and susceptibility to false-positive results. Current evidence supports their use as adjuncts to cystoscopy and cytology rather than replacements. These limitations have driven the development of mutation-based, epigenetic, and sequencing-based urine assays discussed in subsequent sections.

## 5. Emerging Urinary Biomarkers Not Yet Approved for Routine Clinical Use

While FDA-approved urinary biomarkers have improved detection compared with cytology, their limited specificity and modest clinical impact have driven the development of molecular assays targeting tumor-specific genetic and epigenetic alterations. These emerging approaches aim to directly detect oncogenic events characteristic of urothelial carcinoma (UC), thereby improving sensitivity, enabling earlier detection, and supporting longitudinal disease monitoring. Mutation-based, epigenetic, and sequencing-based assays increasingly form the foundation of urine-based liquid biopsy strategies [28]. Although most remain investigational, accumulating evidence supports their potential clinical utility, particularly in surveillance and risk-adapted management.

### 5.1. FGFR3 Mutation-Based Assays

Fibroblast growth factor receptor 3 (FGFR3) is a receptor tyrosine kinase frequently altered in UC. Activating FGFR3 mutations occur in approximately 40–60% of low-grade, NMIBC and are uncommon in high-grade or muscle-invasive disease [29]. These mutations are associated with favorable prognosis, including lower progression risk and improved cancer-specific survival. Urine-based detection of FGFR3 mutations exploits their high prevalence and temporal stability [30]. Targeted assays using allele-specific PCR or ultra-deep amplicon sequencing have demonstrated sensitivities ranging from 50% to 60% with near-perfect specificity. When incorporated into multianalyte panels combining FGFR3 mutation detection with protein biomarkers and DNA methylation markers, diagnostic performance improves substantially. For example, combined assays have achieved positive predictive values exceeding 90% and negative predictive values above 95%, with minimal interference from hematuria or inflammation. Despite these promising results, FGFR3-based assays are primarily applicable to low-grade NMIBC and are less informative for high-grade tumors, limiting their standalone clinical utility.

### 5.2. TERT Promoter Mutations

Hotspot mutations in the TERT promoter, most commonly C228T (−124 bp) and C250T (−146 bp), represent one of the most frequent genetic alterations in UC, occurring in approximately 60–80% of tumors across all stages and grades [31,32]. These mutations create de novo binding sites for ETS transcription factors, leading to telomerase reactivation and cellular immortalization. Importantly, TERT promoter mutations are considered early (founder) events and tend to persist throughout tumor progression and recurrence, making them attractive biomarkers for longitudinal monitoring. Urinary detection of TERT promoter mutations using digital droplet PCR (ddPCR), allele-specific qPCR, or NGS has demonstrated sensitivities of approximately 70–85% and specificities exceeding 90% for primary diagnosis, with even higher performance reported in surveillance settings. Several studies have shown that urinary TERT promoter mutations outperform cytology in low-grade disease and may detect molecular recurrence months before cystoscopic evidence becomes apparent. However, reduced assay performance has been reported in samples with marked inflammation or low tumor DNA fraction, underscoring the need for standardized pre-analytical workflows.

### 5.3. Epigenetic and DNA Methylation Assays

Epigenetic alterations, particularly aberrant DNA methylation, play a central role in urothelial carcinogenesis. Hypermethylation of tumor suppressor gene promoters can be detected in urine and has been extensively investigated as a diagnostic and surveillance biomarker [33]. Panels combining multiple methylation markers have demonstrated higher sensitivity than cytology, especially for low-grade tumors, with reported sensitivities ranging from 70% to 90% [34]. However, specificity is often reduced, with false-positive results observed in benign inflammatory conditions, urinary tract infections, and after recent instrumentation [34]. Heterogeneity in assay design, marker selection, and cutoff thresholds has limited cross-study comparability and guideline endorsement. As a result, methylation-based assays remain investigational, though they are frequently incorporated into multianalyte testing strategies.

### 5.4. Next-Generation Sequencing (NGS) of Urine

NGS has enabled comprehensive genomic profiling of UC using urine as a non-invasive biospecimen. Targeted NGS panels typically interrogate recurrently altered genes such as FGFR3, TP53, PIK3CA, ERBB2, and TERT, as well as copy number alterations and mutational signatures [35]. NGS can be applied to DNA extracted from exfoliated cells, urinary sediment, or cell-free fractions. Multiple studies have demonstrated high concordance between genomic alterations detected in urine and matched tumor tissue [36]. Importantly, NGS-based assays can detect minimal residual disease and molecular recurrence months before clinical or cystoscopic relapse [35]. Despite their high analytical sensitivity and breadth, NGS assays face barriers to routine implementation, including higher cost, longer turnaround times, and substantial bioinformatic requirements. Pre-analytical variables—such as urine volume, preservation methods, and DNA extraction protocols—significantly influence assay performance and require standardization [37].

### 5.5. Urinary Cell-Free DNA (cfDNA)

Urinary cell-free DNA (cfDNA) consists of short DNA fragments released through apoptosis, necrosis, or active secretion. In UC, tumor-derived cfDNA is shed directly into urine, often resulting in higher tumor fractions than observed in plasma [38]. Analysis of urinary cfDNA enables detection of point mutations, copy number alterations, and methylation patterns, supporting applications in diagnosis, surveillance, and treatment monitoring. Several studies have shown that urinary cfDNA can identify recurrence earlier than cystoscopy and predict disease progression. However, urinary cfDNA analysis is technically challenging. Variable contamination with genomic DNA from lysed urothelial cells, along with sensitivity to storage conditions and processing delays, can dilute tumor-derived signals. Development of standardized collection and processing protocols remains essential for clinical translation.

### 5.6. Exosomes and Extracellular Vesicles

Exosomes and other extracellular vesicles are membrane-bound particles released by cells and enriched with proteins, lipids, DNA, mRNA, and non-coding RNAs reflective of their cell of origin [39]. Tumor-derived exosomes, being a biologically rich source of tumor-derived nucleic acids and proteins, are abundant in urine and protected from enzymatic degradation, making them attractive biomarker carriers [40]. Proteomic and transcriptomic profiling of urinary exosomes has identified candidate signatures associated with UC diagnosis, stage, and prognosis. However, significant methodological variability exists, including differences in isolation methods (ultracentrifugation vs. commercial kits), pre-analytical handling, quantification platforms, and normalization strategies [41]. These challenges currently limit reproducibility and hinder clinical implementation.

### 5.7. MicroRNAs and Other Non-Coding RNAs

MicroRNAs (miRNAs) regulate gene expression post-transcriptionally and are dysregulated in UC. Urinary miRNAs are stable and detectable either freely or within extracellular vesicles. Numerous studies have reported diagnostic and prognostic miRNA panels capable of distinguishing UC patients from controls and predicting recurrence [42]. Several urinary microRNAs have emerged as promising diagnostic and prognostic biomarkers in UC. Frequently reported candidates include miR-126 [43] and miR-145, which are associated with tumor suppression, as well as oncogenic miRNAs such as miR-21 and members of the miR-200 family. Panels combining multiple miRNAs have demonstrated improved diagnostic accuracy compared with single markers, although external validation and standardization remain limited [44]. Beyond miRNAs, long non-coding RNAs and circular RNAs are emerging as potential urinary biomarkers. While preliminary results are promising, the lack of standardized detection platforms and validation cohorts has limited clinical adoption [45].

## 6. Screening, Diagnosis, and Surveillance: Clinical Context of Urine-Based Testing

Urine-based diagnostics in urothelial carcinoma (UC) are applied in three distinct clinical contexts—screening, diagnosis, and surveillance—each with different biological constraints and performance requirements [46]. Failure to distinguish between these settings has contributed to inconsistent interpretation of biomarker performance across studies.

Routine screening is not common practice in the general, asymptomatic population. In practice, screening most often occurs as part of the workup of hematuria (gross or microscopic), irritative urinary symptoms, or recurrent UTIs, as well as through targeted evaluation of selected high-risk individuals (e.g., carriers of hereditary cancer syndromes, individuals with a heavy smoking history, significant occupational exposure to aromatic amines, prior pelvic radiation, cyclophosphamide exposure, some chronic inflammatory bladder conditions) [47,48]. In this setting, high sensitivity and negative predictive value are essential to minimize missed cancers. However, the relatively low prevalence of UC in most screened populations limits the clinical utility of even highly sensitive assays, as false-positive results may lead to unnecessary invasive procedures. Consequently, urine cytology has limited screening value, and molecular assays remain largely investigational, typically evaluated only in enriched high-risk cohorts [49].

Diagnosis refers to the evaluation of patients presenting with hematuria or other symptoms suggestive of UC. Here, urine-based tests function as adjuncts to cystoscopy and imaging, aiming to improve the detection of carcinoma in situ (CIS), high-grade disease, or lesions not readily visible endoscopically. Balanced sensitivity and specificity are required, and biomarkers detecting tumor-specific genomic alterations, such as TERT promoter mutations or copy number changes, may provide incremental diagnostic value beyond cytology, particularly for high-grade tumors [50].

Surveillance represents the most established and clinically impactful application of urine-based diagnostics, particularly in NMIBC. Surveillance assays prioritize high sensitivity, reproducibility, and longitudinal consistency to enable early detection of recurrence and potentially reduce cystoscopy frequency. Serial urine testing allows patient-specific baseline comparisons, increasing the clinical relevance of molecular signals compared with single time-point testing [28,50]. In this context, urinary cfDNA and targeted NGS panels are particularly well suited for monitoring molecular recurrence.

## 7. NMIBC Versus MIBC: Biological Context and Biomarker Implications

The distinction between NMIBC and MIBC is fundamental for understanding the strengths and limitations of urine-based diagnostics. These disease entities arise through partially divergent molecular pathways and exhibit different patterns of tumor shedding into urine [6,51]. NMIBC is commonly driven by activating mutations in FGFR3, HRAS, and PIK3CA and is characterized by relatively stable genomes and lower mutational burden [52,53]. Low-grade papillary tumors often shed limited numbers of tumor cells and small quantities of tumor-derived DNA, resulting in poor sensitivity of urinary cytology and low cfDNA abundance. Consequently, detection of NMIBC often relies on highly sensitive, targeted assays directed at recurrent driver mutations, particularly FGFR3 and TERT promoter alterations [50,54]. In contrast, MIBC is characterized by genomic instability, frequent alterations in TP53, RB1, ERCC2, and chromatin remodeling genes, as well as higher tumor mutational burden and copy number alterations [7,55]. Increased cell turnover, necrosis, and stromal invasion lead to greater release of tumor-derived DNA into urine. As a result, urinary cfDNA concentration and fragment size profiles are more robust in MIBC, improving the performance of NGS-based assays and enabling broader genomic profiling [35,36]. These biological differences underscore why a single urine-based assay is unlikely to be optimal across disease stages. Stage-adapted biomarker strategies, integrating cytology with mutation-based or cfDNA-based assays, are therefore more likely to achieve clinically meaningful performance.

## 8. Upper Urinary Tract Urothelial Carcinoma (UTUC): Diagnostic Challenges

Upper urinary tract urothelial carcinoma (UTUC) presents unique anatomical and biological challenges for urine-based diagnostics. Compared with bladder cancer, UTUC is less common and often diagnosed at more advanced stages, partly due to limitations in early detection [56,57]. Selective upper tract urine cytology demonstrates high specificity but variable sensitivity, particularly for low-grade tumors. Sampling limitations, dilution effects, and intermittent tumor shedding contribute to false-negative results [58,59]. Nevertheless, cytology remains a cornerstone for detecting high-grade UTUC and CIS when combined with imaging and ureteroscopy. Targeted NGS analysis of urine—either voided or selectively collected—has shown promise in UTUC, reflecting the high prevalence of recurrent mutations such as FGFR3, TERT promoter, KMT2D, and TP53 [60,61]. Molecular assays can improve sensitivity over cytology, particularly when selective upper tract sampling is feasible. However, distinguishing UTUC-derived mutations from concomitant or prior bladder tumors remains challenging, especially in patients with multifocal disease [62,63]. Urinary cfDNA analysis in UTUC is influenced by anatomical dilution, as tumor-derived DNA must traverse the renal collecting system before voiding. Renal function, urine flow, tumor size, and location all affect cfDNA concentration and detectability. Despite these limitations, cfDNA-based assays may be particularly valuable in post-nephroureterectomy surveillance, where detection of tumor-specific mutations could signal bladder recurrence or residual upper tract disease. In UTUC, anatomical dilution of tumor-derived DNA limits the sensitivity of voided urine assays. Selective upper-tract urine collection via ureteral catheterization has been shown to improve the detection rates of methylation-based and NGS assays, supporting its use in high-risk or diagnostically challenging cases [64].

## 9. Integrative and Multimodal Approaches in Urine-Based Diagnostics

No single urine-based assay currently achieves sufficient diagnostic accuracy to replace cystoscopy across all clinical settings. Consequently, integrative and multimodal approaches combining cytology with molecular biomarkers have gained increasing attention [46,65]. These strategies aim to leverage the high specificity of cytology for high-grade disease and the improved sensitivity of molecular assays for low-grade or early-stage tumors.

Multimodal testing approaches may integrate cytology with mutation-based assays, epigenetic markers, or targeted NGS panels. Such combinations have demonstrated improved overall diagnostic performance compared with individual assays, particularly in surveillance of NMIBC [57,66]. Longitudinal assessment further enhances clinical utility, as patient-specific baseline molecular profiles allow detection of dynamic changes indicative of recurrence or progression.

Advances in computational analysis, including machine learning and artificial intelligence, enable integration of heterogeneous biomarker data into predictive models. Early studies suggest that these approaches may improve risk stratification and facilitate personalized surveillance schedules, potentially reducing unnecessary cystoscopies while maintaining oncological safety [67,68]. However, most multimodal algorithms remain in early development and require prospective validation before routine clinical adoption.

Across urothelial carcinoma subtypes and clinical contexts, urine-based diagnostics are transitioning from morphology-based assessment toward genomics-driven, longitudinal monitoring tools. Optimal application depends on disease stage (NMIBC vs. MIBC), anatomical site (bladder vs. upper urinary tract), and clinical intent (screening, diagnosis, or surveillance). Risk-adapted strategies integrating cytology with targeted molecular assays are therefore most likely to achieve meaningful clinical impact.

## 10. Study Heterogeneity

Interpretation of diagnostic performance across urine-based assays is complicated by substantial heterogeneity among studies. Differences in patient populations (screening vs. surveillance cohorts), disease prevalence, tumor grade distribution, reference standards, and clinical endpoints contribute to wide variability in reported sensitivity and specificity [46,69]. These factors must be carefully considered when comparing assays and translating results into clinical practice.

## 11. Clinical Implementation, Challenges, and Future Perspectives

Despite substantial technological advances, several barriers continue to limit the routine clinical implementation of urine-based diagnostics. Key challenges include lack of assay standardization, variability in pre-analytical and analytical workflows, limited prospective validation, and uncertainty regarding clinically actionable thresholds [70,71]. Cost considerations, reimbursement policies, and access to specialized laboratory infrastructure further influence adoption.

Regulatory approval and guideline endorsement require robust evidence demonstrating not only analytical validity but also clinical utility, cost-effectiveness, and impact on patient outcomes. To date, most urinary biomarkers have demonstrated incremental diagnostic value but have not consistently shown the ability to safely replace cystoscopy in large prospective trials [16,72]. As a result, major professional societies continue to recommend urine-based biomarkers primarily as adjuncts rather than standalone diagnostic tools.

While urine-based molecular assays offer the potential to reduce cystoscopy frequency, their adoption is strongly influenced by cost, laboratory infrastructure, and reimbursement. Genomic profiling assays are generally more expensive than cytology and may require specialized equipment, whereas cystoscopy remains widely available despite its invasiveness. Emerging health-economic analyses suggest that biomarker-guided surveillance strategies may become cost-effective in selected risk groups [73,74].

Future directions in urothelial carcinoma management are likely to involve personalized, risk-adapted surveillance strategies incorporating urine-based liquid biopsy. Continued improvements in sequencing technologies, assay sensitivity, and bioinformatic analysis may enable cystoscopy-sparing algorithms for selected patient populations, particularly those with low- or intermediate-risk NMIBC [75,76]. Integration of genomic, epigenetic, and transcriptomic data into unified diagnostic platforms represents a key area of ongoing research.

NCT03973307 study aims to test if the UroX™ biomarker to assess its value as a screening tool for bladder cancer [77]. NCT06126796 evaluates patient-reported preference for urine based molecular testing (CxBladder Monitor) compared to cystoscopy for patients on surveillance for NMIBC [78].

Large prospective trials, real-world implementation studies, and cost-effectiveness analyses will be essential to define the role of urine-based diagnostics in precision oncology. As these technologies mature, they hold the potential to reduce patient burden, improve quality of life, and enable earlier therapeutic intervention while maintaining oncological safety [4,79]. Future research should prioritize prospectively designed trials evaluating urine-based biomarkers in real-world clinical pathways. Ongoing studies incorporating NGS, cfDNA, methylation, and multi-omics approaches are expected to clarify their role in cystoscopy-sparing surveillance and early detection strategies [4,80].

## Figures and Tables

**Table 1:** NMIBC vs. MIBC: Biomarker and cfDNA Characteristics.

Feature	NMIBC	MIBC
Common driver alterations	FGFR3, HRAS, PIK3CA, TERT promoter	TP53, RB1, ERCC2, chromatin remodeling genes
Genomic stability	Relatively stable	Highly unstable
Tumor mutational burden	Low–moderate	High
Tumor DNA shedding into urine	Low	High
cfDNA abundance	Often low, near detection limits	Elevated
Best-suited urine assay type	Targeted mutation panels	Broad NGS panels, cfDNA profiling
Utility of cytology	Limited (except CIS)	High

**Table 2:** Most Frequent Mutations in UC.

Gene	Approx. Frequency	Functional Category	Notes
*TP53*	40–60% (More frequent in MIBC)	Tumor suppressor, Cell cycle, DNA damage	Genomic instability, aggressive disease
*TERT promoter*	60–80% (all stages)	Telomerase activation	Early event, diagnostic urine marker
*KDM6A*	30–45%	Epigenetic regulator, chromatin remodeling	More common in luminal tumors
*ARID1A*	25–40%	Chromatin remodeling (SWI/SNF)	Often co-mutated with KDM6A
Gene	Approx. Frequency	Pathway	Notes
*FGFR3*	15–25% overall (≈40–60% NMIBC)	Receptor Tyrosine Kinase (RTK) signaling/MAPK pathway	Luminal papillary, targetable
*PIK3CA*	20–25%	PI3K–AKT–mTOR	Often with FGFR3
*RB1*	15–25%	Cell cycle	Basal/Squamous, NE-like
*STAG2*	15–25%	Cell division, gene expression and DNA repair	Chromosomal segregation
*EP300/CREBBP*	15–25%	Epigenetic regulation	Transcriptional control
*ERBB2 (HER2)*	5–15%	Receptor Tyrosine Kinase (RTK) signaling	Amplification > mutation (LumU)
*ERBB3*	5–15%	Receptor Tyrosine Kinase (RTK) signaling	Luminal unstable
*CDKN2A*	5–15%	Cell cycle	Often homozygous deletion
*TSC1/TSC2*	5–15%	mTOR	Sensitivity to mTOR inhibition
*ATM/BRCA1/BRCA2/ERCC2*	5–15%	DNA damage repair	Platinum sensitivity (esp. ERCC2)
*N/H/KRAS*	5–15%	MAPK	More common in NMIBC

**Table 3:** Summary of the 2020 Consensus Molecular Classification of MIBC.

Consensus Subtype	Key Biological/Molecular Features	Common Genomic Alterations	Microenvironment
Luminal Papillary (LumP) (≈24%)	Luminal differentiation; enriched urothelial markers (PPARG, GATA3, FOXA1)	FGFR3 mutations/amplifications frequent; KDM6A mutations; CDKN2A deletion frequent; FGFR3-associated transcriptional activity high	Less immune infiltration; papillary pathway features; potential FGFR3-targeted therapy relevance
Luminal Non-specified (LumNS) (≈8%)	Luminal phenotype with stromal/immune infiltration	Elevated stromal and immune signals; PPARG signature present	Higher B-cell and T-cell infiltration compared with other luminal subtypes
Luminal Unstable (LumU) (≈15%)	Luminal markers with high cell-cycle activity	TP53 mutations common; ERBB2/ERBB3 amplifications; high PPARG expression; high somatic mutation burden	Genomic instability and active proliferation
Stroma-rich (≈15%)	Mesenchymal features dominate	Not driven by a single dominant oncogene	Strong stromal cell signatures (fibroblasts, smooth muscle); distinct immune infiltrates (T cells, B cells)
Basal/Squamous (Ba/Sq) (≈35%)	Basal and squamous differentiation; basal markers (cytokeratins)	TP53 mutations frequent; RB1 alterations; often low PPARG/GATA3	Enriched cytotoxic lymphocytes and NK cells; may share features with squamous tumors across cancer types
Neuroendocrine-like (NE-like) (≈3%)	Neuroendocrine gene expression	Often TP53 and RB1 co-mutated in similar contexts	Minimal immune infiltration

**Table 4:** Urine Biomarkers for Screening, Diagnosis, and Surveillance of Urothelial Carcinoma.

Biomarker/Test	Biomarker Type	Detection Method	Clinical Use	Strengths	Limitations
Urine cytology	Cellular morphology	Microscopy	Diagnosis, surveillance	High specificity, especially for high-grade tumors	Low sensitivity for low-grade disease, operator-dependent
NMP22	Nuclear matrix protein	Immunoassay	Diagnosis, surveillance	Easy to perform, point-of-care versions available	High false-positive rate in benign conditions
BTA stat/BTA TRAK	Complement factor H–related protein	Immunoassay	Diagnosis, surveillance	Rapid results	Low specificity, affected by hematuria and inflammation
uCyt+ (formerly ImmunoCyt)	Detects mucins and CEA	Fluorescent IHC assay	Monitoring patients for recurrence, particularly those with low-risk disease	High sensitivity for detecting UC of all grades and stages, including CIS	Specificity is generally lower than cytology alone
UroVysion FISH	Chromosomal aneuploidy (3, 7, 17, 9p21)	Fluorescence in situ hybridization	Diagnosis, surveillance	Higher sensitivity than cytology, useful for equivocal cases	Costly, requires specialized laboratory
Cxbladder	mRNA expression panel	RT-PCR	Surveillance, triage	High sensitivity, strong negative predictive value	Limited availability, cost considerations
DNA methylation assays	Epigenetic alterations	PCR-based methods	Early detection, surveillance	High diagnostic accuracy, non-invasive	Lack of standardization, limited clinical adoption
Urine tumor DNA (utDNA)	Somatic mutations/aneuploidy	Next-generation sequencing	Detection, surveillance	Enables molecular monitoring and risk stratification	High cost, technical complexity
microRNAs	Regulatory RNA molecules	RT-PCR	Experimental diagnosis	Stability in urine, promising accuracy	Mostly investigational, heterogeneous results
Urinary exosomes	Protein/RNA cargo	Proteomics, RNA analysis	Experimental biomarkers	Reflect tumor biology	Isolation challenges, lack of validation

**Table 5:** Performance Considerations by Clinical Use Case.

Clinical context	Urine Cytology	Commercial Urine Tests	NGS/cfDNA Assays
Screening (high-risk populations)	Limited utility due to low sensitivity	Investigational; false positives limit broad screening	Investigational; best suited for enriched cohorts
Initial diagnosis (hematuria workup)	Adjunct to cystoscopy; strong for high-grade disease	Adjunctive; may increase detection vs. cytology alone	Adjunctive; improves detection of CIS and occult tumors
NMIBC surveillance	Low sensitivity for recurrence	Used to reduce cystoscopy frequency	High potential to reduce cystoscopy burden via serial testing
MIBC detection	Detects high-grade disease	Moderate performance	Strong performance due to higher cfDNA shedding
Post-treatment monitoring	Limited	Moderate	Strong (molecular residual disease, early recurrence)

## Data Availability

No new data were created or analyzed in this study. Data sharing is not applicable to this article.

## List of Abbreviations

CIS	carcinioma in situ
UC	urothelial carcinoma
HGUC	high-grade urothelial carcinoma
LGUC	low-grade urothelial carcinoma
BC	bladder cancer
TPS	Paris System for Reporting Urinary Cytology
CfDNA	cell free DNA
NGS	next-generation sequencing
NMP22	nuclear matrix protein 22
BTA	bladder tumor antigen
UTUC	upper urinary tract urothelial carcinoma
